# First Strike personalized predictive radioiodine prescription for inoperable metastatic differentiated thyroid cancer

**Published:** 2023

**Authors:** Yung Hsiang Kao

**Affiliations:** Department of Nuclear Medicine, The Royal Melbourne Hospital, Australia

**Keywords:** Radioiodine, Differentiated thyroid cance, Dosimetry, Marrow, Dose rate, Theranostics

## Abstract

**Objective(s):**

The traditional practice of empiric radioiodine (I-131) prescription is scientifically obsolete and inappropriate for inoperable metastatic differentiated thyroid cancer. However, theranostically guided prescription is still years away for many institutions. A personalized predictive method of radioiodine prescription that bridges the gap between empiric and theranostic methods is presented. It is an adaptation of the “maximum tolerated activity” method, where serial blood sampling is replaced by population kinetics carefully chosen by the user. It aims to maximize crossfire benefits within safety constraints to overcome tumour absorbed dose heterogeneity for a safe and effective first radioiodine fraction i.e., the First Strike.

**Methods:**

The EANM method of blood dosimetry was incorporated with population kinetics, marrow and lung safety constraints, body habitus and clinical assessment of metastatic extent. Population data of whole body and blood kinetics in patients with and without metastases, prepared by recombinant human thyroid stimulating hormone or thyroid hormone withdrawal, and the maximum safe marrow dose rate were deduced from published data. For diffuse lung metastases, the lung safety limit was linearly scaled by height and separated into lung and remainder-of-body components.

**Results:**

The slowest whole body Time Integrated Activity Coefficient (TIAC) amongst patients with any metastases was 33.5±17.0 h and the highest percentage of whole body TIAC attributed to blood was 16.6±7.9%, prepared by thyroid hormone withdrawal. A variety of other average radioiodine kinetics is tabulated. Maximum safe marrow dose rate was deduced to be 0.265 Gy/h per fraction, where blood TIAC is normalised to administered activity. An easy-to-use calculator was developed which only requires height, weight and gender to populate recommendations for personalized First Strike prescription. The user decides by clinical gestalt whether the prescription is to be constrained by marrow or lung, then selects an activity depending on how extensive the metastases are likely to be. A Standard Female with oligometastasis and good urine output without diffuse lung metastasis is expected to safely tolerate 8.03 GBq of radioiodine as the First Strike.

**Conclusion:**

This predictive method will help institutions rationalise the First Strike prescription based on radiobiologically sound principles, personalised to individual circumstances.

## Introduction

 For inoperable metastatic differentiated thyroid cancer, the traditional practice of empiric radioiodine (I-131) prescription is scientifically obsolete and conceptually incompatible with modern personalized oncology (1-2). 

 Fundamentally, I-131 therapy is continuous low dose rate molecular radiotherapy, where tissue effects follow predictable deterministic radiobiology (3). Each administration of I-131 is a fraction of molecular radiotherapy that should be prescribed based on radiobiologic principles to ensure safety and efficacy. I-131 should not be prescribed like a common medicine (2, 3).

 More than half a century of I-131 literature, including those published by institutions which prescribe up to “maximum tolerated activity”, only considers safety, not tumour efficacy (2, 3). 

 Tumour absorbed doses were not, or in most cases, could not be calculated in the vast majority of published I-131 studies due to lack of tools, resources or standardised methods. Without knowing tumour absorbed doses, data cannot be appropriately stratified for meaningful outcomes analysis amongst other parameters (2, 3).

 The concept of the “First Strike”, i.e., the first I-131 fraction, stems from decades of clinical observation that the first fraction confers the greatest therapeutic benefit, whereas subsequent fractions become progressively less effective (4-5). Mechanistically, this observation may be explained by natural internal heterogeneity within every tumour nodule, containing regions of differing cellular iodine avidity which become worse after each I-131 fraction due to survival of less iodine-avid cells. Therefore, tumour intra- and inter-lesional heterogeneity is an ever-present, unavoidable problem that must be considered in every patient with metastasis (2).

 I-131 emits low energy beta particles with a short mean tissue range of approximately 0.4 mm (mean beta energy 192 keV) and maximum tissue range of approximately 3 mm (maximum beta energy 807 keV) (6). Mechanistically, an effective First Strike overcomes the natural internal heterogeneity of non-uniform intra-tumoral absorbed dose biodistribution by maximizing crossfire from iodine-avid cells to adjacent poorly (or non-) iodine avid cells, within limits of the short path length of I-131 beta particles. However, crossfire cannot reach non-iodine avid tumour cells situated beyond the maximum I-131 path length of the nearest iodine-avid cell. This explains why micrometastases have better response rates than bulky bone metastases (5).

 Nevertheless, to maximize crossfire benefits to non-iodine avid tumour cells within range of I-131 beta particle path length, tumour absorbed dose (Gy) prescription has no upper limit and should ideally exceed 100 Gy (2). The final prescription is constrained by either normal tissue tolerance (usually marrow or lung) or radiation exposure levels permitted by local regulatory licensing. Predictive prescription constrained by normal tissue is the radiobiologic principle underlying the established method of “maximum tolerated activity” prescription, currently practised in some theranostic institutions today (5). Its apparent lack of clinical success is explained by insidious critical flaws in data analysis unbeknownst to most, which has unfortunately misled generations of nuclear medicine specialists (2, 3).

 No tumour is ever truly radioresistant (2). The benefit of crossfire is greatest when there is a large number of iodine-avid cells to cross-irradiate adjacent poorly iodine-avid tumour cells i.e., the First Strike is the most important. Non-iodine avid tumour cells beyond the maximum I-131 path length will remain untreated by crossfire and may require other adjuvant modalities. After the first fraction, iodine-avid tumour cells are mostly eliminated leaving behind weakly iodine-avid tumour cells which respond poorly and become progressively worse after subsequent fractions (4-5). Conceptually, this so-called “radioresistance” is arguably an iatrogenic problem caused by inadequate First and subsequent Strikes, bred by repeated exposure to fractionated sub-tumoricidal irradiation. I-131 fractionation is therefore a double-edged sword that requires a fine balance between safety and tumour efficacy. If the aim is to cure metastatic disease, fractionation should be minimized as it may attenuate tumour response. If the aim is palliation (e.g., elderly), then fractionation would be relatively beneficial to avoid radiotoxicity.

 The First Strike is an ideal solution to overcome the natural problem of intra-tumoral absorbed dose heterogeneity, available today in theranostic institutions practicing “maximum tolerated activity” predictive prescription. We define “theranostics” as the qualitative and quantitative analysis of a diagnostic-therapeutic pair, taking into consideration radiobiology and patient-specific radiomics (e.g., radionuclide kinetics) extracted from a simulation study to optimise predicted radiation absorbed doses (Gy) when formulating the activity prescription (Bq). In I-131 theranostics, patient-specific kinetics are simulated using a surrogate such as I-123, I-124 or very low activity I-131. This allows for personalized predictive prescription by balancing normal tissue tolerance with tumour efficacy. However, theranostically prescribed “maximum tolerated activity” requires expertise and resources beyond reach of many institutions today. Simple semi-empiric prescription based on MBq/kg or Gy/MBq have been described (5). However, such composite metrics are only suitable for broad generalizations and unsuitable to apply to an individual patient. 

 Moreover, a male vs female patient with the same metastatic burden will exhibit different I-131 kinetics due to differences in body habitus and physiology. It is also possible for two patients of the same gender and metastatic burden to have different I-131 kinetics. In other words, empiric prescription is inherently radiobiologically inadequate to balance individual case complexities. Furthermore, traditional archaic increments of 1 to 2 GBq begs the question why I-131 prescription cannot be scientifically rationalised down to a single Becquerel, as is standard-of-care for yttrium-90 radioembolization (2).

 A novel method of First Strike personalised predictive prescription is presented here, derived from meta-analyses of published I-131 population kinetics. It is conceptually superior to simple composite metrics (e.g., MBq/kg or Gy/MBq) by breaking up its dosimetric parameters into individual components for separate, personalised and rational consideration, within safety limits of assumed population kinetics. It is an adaptation of the “maximum tolerated activity” method, where resource intensive serial blood sampling and dosimetry is replaced by population kinetics carefully chosen by the user. This is a transitionary method to bridge the paradigm gap from empiric to theranostic prescription.

## Predictive schema

***Clinical pre-requisites and limitations***

 An effective First Strike requires total thyroidectomy with minimal thyroid remnants. Large thyroid remnants will consume I-131, rendering the First Strike ineffective to treat metastatic disease. Institutions where 24 h I-131 neck uptake is consistently <1% (i.e., “excellent” resection) are ready to implement the First Strike principle (7). Other clinical pre-requisites are: adult patients, normal full blood count, low iodine diet, orally administered I-131 (iodide), no chronic lung disease or advanced chronic kidney disease, well hydrated with good urine output and bowel motion. Be cautious with the elderly, renal impairment and extremes of body habitus as their I-131 kinetics could fall beyond average population assumptions. Invalid in end-stage kidney disease and paediatrics. Abscopal effects are unknown and assumed negligible.

 The First Strike is for patients with inoperable iodine-avid metastatic differentiated thyroid cancer. It is not relevant for remnant ablation or operable metastases. Metastatic extent may be known or clinically suspected. This may be clinically assimilated from intra-operative findings, histopathologic features of aggressiveness, >6-week post-thyroidectomy thyroglobulin levels or any non-iodine imaging (e.g., non-contrast CT, MRI, FDG PET/CT). A normal CT chest may not fully exclude lung micrometastases, which could be sub-radiologic.

 Our method only applies to patients with unknown I-131 kinetics, a common scenario in institutions practising empiric prescription. If any form of pre-therapy I-131 simulation (e.g., I-123, very low activity I-131 or I-124) including blood kinetics was performed for any reason, it would have revealed the patient-specific kinetics for which the treating team is obligated to analyse theranostically, and therefore the methods here no longer apply. It follows that our method should not be applied to subsequent I-131 fractions (i.e., Second or Third Strikes, etc) because these should be personalised based on patient-specific I-131 kinetics, imaging findings and clinical response analysed after the First Strike.

***Marrow population kinetics***

 A literature review was performed and studies were selected based on whether their published kinetic data were sufficiently detailed to permit meta-analyses. These studies are listed in Supplemental Data. Our institutional kinetic data was also included. Meta-analyses focused on the whole body time-integrated activity coefficient (TIAC; formerly known as Residence Time) and the percentage of whole body TIAC attributed to blood (8). Population averages and its standard deviations (SD) were obtained by simple proportions weighted by cohort size. Kinetic data was separated into recombinant human thyroid stimulating hormone (rhTSH) or thyroid hormone withdrawal (THW) groups, and whether any metastases was present. All TIACs are normalized to the administered I-131 activity (9-10). Our meta-analyses found the slowest whole body TIAC amongst patients with any metastases to be 33.5±17.0 h (Table 1) and the highest percentage of whole body TIAC attributed to blood to be 16.6±7.9% (Table 2), prepared by THW.

**Table 1:** 

**Whole body Time Integrated Activity Coefficient**				
**Preparation**	**Cohort**	**n**	**Mean (h)**	**SD**
rhTSH	All	214	17.6	3.9
THW	All	440	21.9	8.4
rhTSH	Any metastases	14	24.0	13.5
THW	Any metastases	60	28.0	15.1
THW	Bone metastases*	36	33.5	17.0

*Patients with bone metastases may also have soft tissue metastases

**Table 2:** 

**Percentage of whole body Time Integrated Activity Coefficient attributed to Blood**				
**Preparation**	**Cohort**	**n**	**Mean (%)**	**SD**
rhTSH	All	33	13.2	1.2
THW	All	79	14.8	3.7
rhTSH	Any metastases	6	15.2	6.0
THW	Any metastases	12	16.6	7.9
THW	Bone metastases*	8	16.1	6.8

*Patients with bone metastases may also have soft tissue metastases

***Probabilistic assumptions***

 To personalize population kinetic data to an individual patient, it is necessary to invoke the Central Limit Theorem to assume all data to be normally distributed around its mean. Under this assumption, +1SD will encompass 84% of a population (i.e., all possibilities to the left of +1SD) and +2SD will encompass 98% (i.e., all possibilities to the left of +2SD). This practical approach was previously used to rationalize and explain dosimetric data for yttrium-90 radioembolization, and more recently, for I-131 single time point tumour dosimetry (11-12). As the whole body TIAC and percentage of whole body TIAC attributed to blood are related parameters, bivariate normal distribution analysis was performed with an assumed positive correlation coefficient of 0.7, which in general statistics reflects moderate to strong correlation.

***Marrow absorbed dose***

 The European Association of Nuclear Medicine (EANM) method of blood dosimetry is based on the Medical Internal Radiation Dosimetry (MIRD) schema and uses peripheral whole blood as a surrogate for the red marrow (13). The marrow absorbed dose (Gy) per unit administered activity (GBq) is the sum of its beta self-irradiation and whole body gamma contributions as (13):

$\frac{D_{M}}{A_{0}}=61∙\tau_{\mathrm{ml of blood}}+\frac{0.106}{W}∙\tau_{\mathrm{WB}}$ (Equation 1)

 Where $\tau_{\mathrm{ml of blood}}$ is the blood TIAC per millilitre (h), $\tau_{\mathrm{WB}}$ is the whole body TIAC (h), W is the patient’s body weight (kg). 

 The above parameters result in $A_{0}$ that is more in keeping with our own clinical experiences as compared to traditional parameters by Benua et al. (13), which we felt were relatively conservative. Blood volume (ml) is estimated from patient height (cm) and weight (kg) using Retzlaff’s formula for male and female, used by Hanscheid et al. (10) for blood dosimetry. Based on prior work by Thomas et al. (9) and Hanscheid et al. (10), $\tau_{\mathrm{ml of blood}}$ may be re-expressed in terms of $\tau_{\mathrm{WB}}$ as:

$\tau_{\mathrm{ml of blood}}=\frac{f∙\tau_{\mathrm{WB}}}{\mathrm{Blood Volume}}$ (Equation 2)

Where f is the fraction of $\tau_{\mathrm{WB}}$ attributed to blood and blood volume is in millilitres (ml).

 Where the likelihood of extensive soft tissue and/or bone metastases (excluding lung) has been clinically assessed to be low, we apply the following THW population kinetics based on Tables 1 and 2: whole body TIAC 50.5h (i.e., 33.5h +1SD 17.0 h) and percentage of whole body TIAC attributed to blood 24.5% (i.e., 16.6% +1SD 7.9%). If the clinical likelihood is medium, we apply whole body TIAC 67.5h (i.e., 33.5h +2SD 34.0 h) and percentage of whole body TIAC attributed to blood 24.5%. If the clinical likelihood is high, we apply whole body TIAC 67.5 h and percentage of whole body TIAC attributed to blood 32.4% (i.e., 16.6% +2SD 15.8%). These assumed population kinetics in conjunction with the marrow dose rate constraint (see next section) are conservative and generally err in favour of safety.

***Marrow absorbed dose rate***

 The absorbed dose rate (Gy/h) is a critical radiobiologic parameter that relates to repair and normal tissue radiotoxicity (14). Schumann et al. (15) showed in an ex-vivo study of peripheral blood mononuclear cells that DNA repair was efficient after irradiation by I-131 at a dose rate of 0.05 Gy/h. However, in-vivo maximum safe I-131 dose rate is yet to be properly defined. In the absence of established human data, it would be prudent to derive an estimate from published real-world data. We draw on the experiences of Deandreis et al. where in their subset of patients >40 years with macrometastases, prepared mostly with rhTSH and dosimetrically prescribed by maximum tolerated activity, their median administered I-131 activity was 10.6 GBq per fraction(16).

 Since most thyroid cancer patients are female, we apply a Standard Female height of 163cm and weight 60kg (17). By meta-analyses of population kinetics, whole body TIAC in metastatic disease by rhTSH was 24.0±13.5 h, and its corresponding percentage of whole body TIAC attributed to blood was 15.2±6.0% (Tables 1 and 2). Since this cohort of patients treated by maximum tolerated activity in the study by Deandreis et al. are from a large experienced tertiary centre, we assume that the metastatic burden of their patients is generally more extensive than average (16). We therefore then take +1SD to encompass 84% of all possibilities to obtain whole body TIAC of 37.5 h and percentage of whole body TIAC attributed to blood of 21.2% i.e., blood TIAC 7.95 h. The above parameters were applied into Equation 1 to obtain the maximum safe marrow absorbed dose of 2.11 Gy per I-131 fraction. The maximum safe marrow dose rate is therefore 2.11÷7.95 = 0.265 Gy/h per fraction, where blood TIAC is normalized to$A_{0}$. This inferred estimate of 0.265 Gy/h is averaged over the duration of blood TIAC. Since blood activity peaks soon after oral I-131 administration, the hourly dose rate in the first few hours will be higher than 0.265 Gy/h, decreasing continuously over time.

 The highest marrow dose rate will be experienced by patients without metastases prepared with rhTSH. From Table 2, the lowest percentage of whole body TIAC attributed to blood (i.e., fastest blood TIAC) was calculated as the mean (13.2%) minus 2SD (2.4%) to obtain 10.8%. Therefore, the parameter f from Equation 2 constrained by dose rate is 0.108. Hence, marrow dose DM (Gy) constrained by marrow dose rate is:

$D_{M}=\mathrm{0.265}∙(f∙\tau_{\mathrm{WB}})$ (Equation 3)

Substituting Equations 2 and 3 into Equation 1 and f=0.108: 

$A_{0}=\frac{0.265∙(f∙\tau_{\mathrm{WB}})}{61∙\left(\frac{0.108∙\tau_{\mathrm{WB}}}{\mathrm{Blood volume}}\right)+\left(\frac{0.106}{W}∙\tau_{\mathrm{WB}}\right)}$ (Equation 4)

Simplified to:

$A_{0}=\frac{0.02862}{\left(\frac{6.588}{\mathrm{Blood volume}}\right)+\left(\frac{0.106}{W}\right)}$ (Equation 5)

 By this method, the maximum safe $A_{0}$ (GBq) constrained by marrow dose rate is only influenced by blood volume (ml) and body weight (kg), independent of $\tau_{\mathrm{WB}}$ (h). 

***Lung tolerance limits***

 If diffuse lung metastasis is known or suspected and is clinically expected to be the main organ affected by metastases, then the dose limiting organ is the lung, not marrow. For safety, guidelines recommend a whole body retained activity of <2.96 GBq at 48 hours in the presence of diffuse lung metastases, based on the early observations by Benua et al (5, 7, 18-19). Implicit in this safety threshold, but not explicitly calculated, is the maximum safe lung dose rate (Gy/h). The method described here is simple and does not replace the more robust method by Sgouros et al. (20). Institutions with such capability are encouraged to do so (21). 

 We choose to linearly scale the lung constraint by patient height, not body weight. This is because lung function may worsen with weight gain and obesity due to mechanical limitations to lung expansion and postulated adipose inflammatory damage (22). This makes scaling by body weight physiologically inappropriate for the lung and therefore scaling by height would be a safer choice. Benua et al. observed lung complications mostly from female patients, and modern dosimetric rationalization was analysed based on a female phantom by Sgouros et al. (20). By assigning the lung tolerance limit to a Standard Female of height 163cm, this limit may be linearly scaled by height to obtain the patient-specific lung constraint (17).

 For simplicity, we assume mono-exponential decay of activities in the lung and in the remainder-of-body. The administered activity $A_{0}$ constrained by lung tolerance is therefore approximately:

$A_{0}=\frac{A_{L_{48}}}{e^{-\lambda_{L}∙48}}+\frac{A_{{\mathrm{RB}}_{48}}}{e^{-\lambda_{\mathrm{RB}}∙48}}$ (Equation 6)

 Where$A_{L_{48}}$is the whole lung activity at 48h, $A_{{\mathrm{RB}}_{48}}$is the remainder-of-body activity at 48h, $\lambda_{L}$and$\lambda_{\mathrm{RB}}$are decay constants for lung and remainder-of-body respectively, calculated as the inverse of their TIACs. 

 To separate the whole body activity at 48 h into its lung and remainder-of-body components, we assume that in the setting of diffuse lung metastases, the percentage of whole body activity within the lung is 80% at 48 h. Lung TIAC is taken from works by Sgouros et al. (20) and Song et al. (21) i.e., 144 h (THW) in diffuse lung metastases and 270 h (rhTSH) in worst-case highly iodine-avid diffuse lung metastases with 90% of whole body activity within the lungs at 48 h. To obtain the remainder-of-body TIAC, we assume that an average amount of bone metastases is present

i.e., 33.5 h (Table 1).

***Predictive calculator***

 Results of population kinetics meta-analyses are summarised in Table 1 and 2. Six datasets including our own cohort were used to calculate the population means and SD for whole body TIAC prepared by rhTSH and THW (23-29). These parameters in patients with metastases were obtained from three datasets including our own cohort (30, 31). 

 Population means and SD for percentage of whole body TIAC attributed to blood were calculated from two studies for both THW and rhTSH (9, 23). These parameters with metastases were obtained from data published by Abuqbeitah et al. (31). Further details of all studies are provided in Supplemental Data. Our tabulated results may be regarded as preliminary; its accuracy is expected to improve as more data becomes available over time.

 For probabilistic interpretation of population kinetic data, bivariate normal distribution analysis was applied to the whole body TIAC and the percentage of whole body TIAC attributed to blood. It showed that +1 SD for both parameters will encompass 77% of all possibilities, +1 SD and +2 SD for each parameter will encompass 84% of all possibilities, and +2 SD for both parameters will encompass 96% of all possibilities.

 Our predictive schema and population kinetics were incorporated into an easy-to-use calculator spreadsheet that is freely provided in Supplemental Data or by contacting the author. The user only needs to key in the patient’s height, weight and gender to populate patient-specific recommendations for First Strike personalized predictive prescription. Our predictive calculator will generate several choices of I-131 activities constrained by marrow and lung where the patient is prepared by THW, not rhTSH. This is because THW generally results in better tumour absorbed doses than rhTSH, therefore THW is preferred for the First Strike unless iatrogenic hypothyroidism is medically or socially contraindicated (3, 32-35). Finally, the user must decide whether the marrow or the lung is the limiting organ, then select an activity based on the clinical likelihood of oligometastasis or extensive metastases.

**Figure 1:**
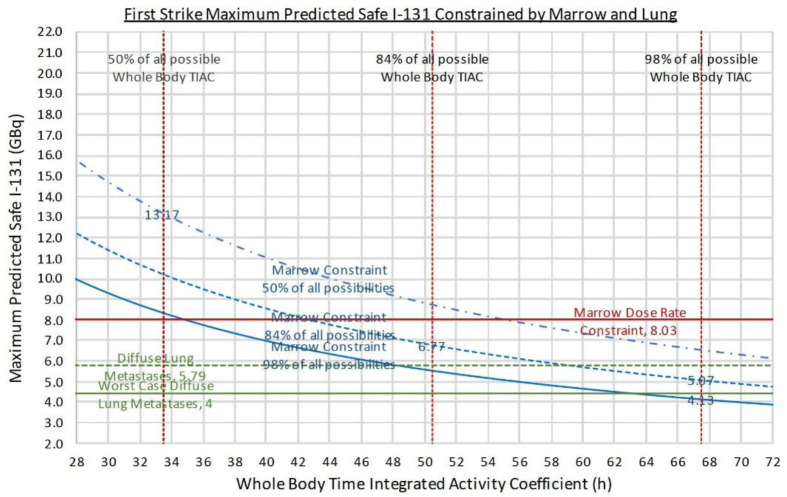
Graphical output generated by our predictive calculator for First Strike I-131 prescription using an example of a Standard Female of height 163cm and weight 60 kg prepared by THW (Table 3). The same results are also presented in the form of a decision tree flowchart to guide clinical decision making (Figure 2). If lungs are the limiting organ, green horizontal lines represent lung limited prescription. If marrow is the limiting organ, the red horizontal solid line represents the prescription constrained by marrow dose rate 0.265 Gy/h, relevant for oligometastasis. The three blue curved lines represent marrow limiting prescription constrained by 2 Gy marrow dose and percentage of total body TIAC attributed to blood at the mean (16.6% i.e., 50% of all possibilities), +1SD (24.5% i.e., 84% of all possibilities) and +2SD (32.4% i.e., 98% of all possibilities). Red vertical dotted lines represent total body TIACs at the mean (33.5h i.e., 50% of all possibilities), +1SD (50.5h i.e., 84% of all possibilities) and +2SD (67.5h i.e., 98% of all possibilities). If extensive soft tissue and/or bone metastases is suspected, suitable marrow limited prescriptions may occur at the intersection of +1 or +2 SD blue curved lines with +1 or +2SD red vertical dotted lines

**Figure 2:**
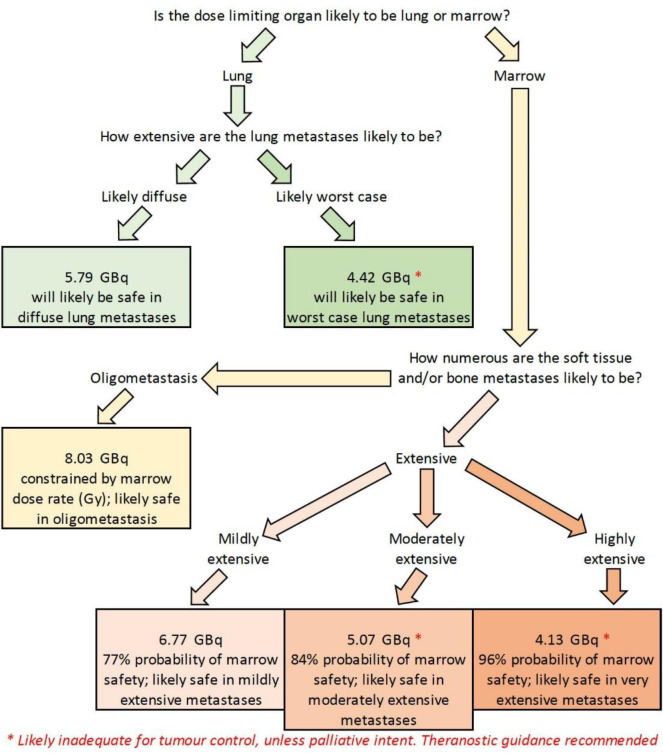
Decision tree flowchart output of the same Standard Female shown in Figure 1, generated by our predictive calculator. This decision tree flowchart presents the same graphical data in a format that is easy to understand for clinical decision making

 Output from our predictive calculator is presented in either graphical format (Figure 1) or decision tree flowchart (Figure 2) to guide clinical decision making. Predictive results of three Standard Man examples are tabulated in Table 3; full results are presented in Supplemental Data. Further technical and clinical guidance on how to interpret our calculator’s predictive prescriptions is provided in Supplemental Data. The radiobiologic rationale for THW to be preferred over rhTSH to treat metastatic disease is also further explained in Supplemental Data.

## Discussion

 Modern theranostics philosophy strives for I-131 to be prescribed correctly the first time, every time, for every patient (1). Semi-empiric metrics based on MBq/kg or Gy/MBq inherently combine four dosimetric variables into one, i.e., whole body TIAC, blood TIAC, blood volume and S value scaling (5, 23, 25, 27). This means that MBq/kg or Gy/MBq metrics are composite results only suitable for broad generalizations, with limited ability to personalize to an individual patient. Our method of First Strike predictive prescription improves the semi-empiric approach by breaking up the four dosimetric components for separate consideration based on population kinetics, height, weight and clinical assessment of metastatic extent.

 Our method is a conceptual simplification of the established method of “maximum tolerated activity” prescription, where resource intensive serial blood sampling and dosimetry is replaced by choices of population kinetics carefully selected by the user. Our method also does not predict any tumour absorbed doses. It is therefore scientifically inferior to both the “maximum tolerated activity” method (i.e., constrained to measured marrow tolerance) and a fully theranostic approach (i.e., both tumour and normal tissue absorbed doses are considered and balanced). However, due to fundamental differences in prescription strategy, all prescription methods (i.e., empiric, semi-empiric, our method, maximum tolerated activity, theranostics) will inevitably result in different prescriptions for the same patient. 

 Philosophically, there is no ‘right’ or ‘wrong’ prescription, but rather, one that is appropriate in the context of each institution’s resources and workflow, balanced with each patient’s clinical circumstances. However, there is a clear movement to encourage institutions still practicing empiric prescription to transition towards modern theranostics (1). 

 Our method assumes that intra-tumoral absorbed dose heterogeneity is severe, therefore prescribes up to the limit of normal tissue tolerance, based on assumed I-131 kinetics, to improve crossfire between adjacent I-131 avid tumour cells. In other words, our method indirectly maximises the tumour absorbed dose and crossfire benefits by constraining the prescribed activity to normal tissue tolerance. Therefore, our method’s primary measure of outcome is safety, whereas tumour response is secondary and patient survival is tertiary.

 However, due to the safety constraints built into our method, patients with moderately or highly extensive metastases may have lower prescriptions that could result in sub-therapeutic tumour absorbed doses (Table 3). 

**Table 3:** 

**First Strike I-131 prescription prepared by THW using Standard Man examples**						
	**Oligometastasis** **(GBq); constrained by marrow dose rate 0.265 Gy/h**	**Extensive soft tissue and/or bone metastases**				
		**Marrow limiting (GBq);** **constrained by 2 Gy marrow dose**			**Lung limiting ** **(GBq)**	
		**Mildly** **extensive**	Moderately* **extensive**	Highly* **extensive**	**Diffuse**	**Worst** case*
Standard Asian Female	7.09	6.07	4.54	3.72	5.68	4.34
Standard Female	8.03	6.77	5.07	4.13	5.79	4.42
Standard Male	10.46	9.08	6.79	5.59	6.25	4.78

* The suggested prescription is likely to be safe but unlikely to achieve an adequate tumour response, unless for palliation; seek theranostic guidance for proper predictive optimisation

 This situation would most likely arise in small females with extensive metastases. Such patients should receive full theranostic guidance, where patient-specific tissue masses and simulated kinetics are meticulously measured to formulate a personalised predictive prescription that balances efficacy with toxicity.

## Conclusion

 An effective First Strike is critical for the optimal treatment of inoperable metastatic differentiated thyroid cancer. Our transitionary method of First Strike personalized predictive I-131 prescription will help institutions bridge the paradigm gap between empiric and theranostic methods. By incorporating population kinetics, body habitus, marrow and lung safety limits, and clinical assessment of metastatic extent, our method can rationalise every single Becquerel of prescribed I-131 activity for each patient, in keeping with best practice standards [1]. Its general principles may be adapted to other forms of systemic radionuclide therapies. Yes, the Holy Gray exists. Learn from modern radioembolization (2).

## Declarations

 Ethics approval and consent to participate: This work was approved by our institutional review board (QA2021112) with waiver of consent.

## Consent for publication

 The author Y.H. Kao consents to this publication.

## Availability of data and material

 All data and materials are available to the reader.

## Competing interests

Y.H. Kao previously received research funding from Genzyme Corporation and Sirtex Medical Limited, and is a proctor for Sirtex Medical Limited.

## Authors' contributions

 Y.H. Kao conceived, researched and wrote this entire work.
